# A Case Study of the Impediments to the Commercialization of Research at the University of Kentucky

**DOI:** 10.12688/f1000research.6487.2

**Published:** 2015-08-04

**Authors:** Nathan L. Vanderford, Elizabeth Marcinkowski

**Affiliations:** 1 Markey Cancer Center and Department of Toxicology and Cancer Biology, College of Medicine, University of Kentucky, Lexington, Kentucky, 40506, USA; 2 Agricultural Biotechnology Program, College of Agriculture, Food and Environment, University of Kentucky, Lexington, Kentucky, 40506, USA

**Keywords:** Research commercialization, entrepreneurship, intellectual property, technology transfer, disclosures, patents, license, start-ups

## Abstract

The commercialization of university-based research occurs to varying degrees between academic institutions. Previous studies have found that multiple barriers can impede the effectiveness and efficiency by which academic research is commercialized. This case study was designed to better understand the impediments to research commercialization at the University of Kentucky via a survey and interview with three successful academic entrepreneurs. The study also garnered insight from the individuals as to how the commercialization process could be improved. Issues with commercialization infrastructure; a lack of emphasis, at the university level, on the importance of research commercialization; a void in an entrepreneurial culture on campus; inhibitory commercialization policies; and a lack of business and commercialization knowledge among faculty were highlighted as the most significant barriers. The research subjects also suggested that commercialization activity may generally increase if a number of factors were mitigated. Such insight can be communicated to the administrative leadership of the commercialization process at the University of Kentucky. Long term, improving university-based research commercialization will allow academic researchers to be more active and successful entrepreneurs such that intellectual property will progress more freely to the marketplace for the benefit of inventors, universities and society.

## Introduction

Research is a vital component of the mission of universities, and indeed academic institutions conduct a substantial volume of research that is funded by government, industry and philanthropic agencies. Development or the commercialization of research should also be a key component of the research mission such that novel ideas, techniques and products can enter the marketplace for the benefit of a variety of stakeholders including inventors, universities and society. In order to facilitate academic-based commercialization, legislation, such as the Bayh-Dole Act, provides universities the legal framework for commercializing the research that is developed within university settings ^1, 2^.

In a commercialization survey conducted by the Association of University Technology Managers (AUTM), in 2013, United States-based institutions generated over 24,000 disclosures, obtained over 5,000 new patents, executed over 5,000 licensing agreements, formed over 800 start-up companies and generated $2.75 billion in license income ^3^. Despite this overall success, academic researchers experience many issues that obstruct the commercialization of research within higher education settings. Previous studies at academic institutions have documented challenges to the commercialization process that include, but are not limited to: risk aversion; constraints on faculty time; lack of financial support; policy/regulation barriers; infrastructure insufficiencies; lack of a common understanding of the value of research commercialization; lack of entrepreneurial thinking among faculty; and lack of interaction and collaboration between universities and industry ^4– 10^. A previous study at the University of Kentucky found that expense, time constraints, insufficient infrastructure and lack of industry partnerships were the most common factors experienced by cancer researchers that impede research commercialization ^11^. Ultimately, challenges to the effective and efficient commercialization of research inhibits obtaining the maximum benefit of university research in that such barriers can prevent university-based innovation from progressing to the marketplace for the benefit of inventors, universities and society.

The University of Kentucky commercializes its research through the Intellectual Property Development and Technology Transfer Office, a unit of the Office of the Vice President for Research. Through this office, the university’s research commercialization activities are historically modest compared to its benchmark institutions. The university currently ranks last among its benchmark institutions in regard to several commercialization metrics including in staffing, invention disclosures, patent applications and license/options executed ( Table 1). And, growth in commercialization activity has been relatively flat from 2010–2013 with the exception of a recent increase in license income ( Table 2). These data could suggest that the University of Kentucky may experience additional commercialization barriers as compared to its benchmark institutions and/or a higher magnitude of common barriers among institutions.

**Table 1.** University of Kentucky research commercialization metrics versus select benchmark institutions (2013) *.

Name of Institution	Research Expenditures	Licensing FTE	Invention Disclosures	Patent Applications	Patents Issued	Licenses and Options Executed	Start-ups	License Income Received
Michigan State University	$515,707,000	5.50	122	49	46	33	1	$3,302,322
Ohio State University	$967,306,055	9.00	384	155	62	50	10	$2,105,127
University of Arizona	$629,466,000	8.50	144	76	27	48	3	$926,023
University of Florida	$544,936,847	13.50	335	152	107	140	16	$28,067,988
University of Iowa	$435,377,000	6.00	96	53	24	29	6	$1,205,342
**University of Kentucky**	**$239,715,000**	**2.00**	**58**	**17**	**30**	**9**	**3**	**$4,800,000**
University of Michigan	$1,328,721,165	9.00	412	148	128	108	9	$14,464,565
University of Minnesota	$882,022,000	18.00	331	148	64	91	14	$38,030,470
University of North Carolina, Chapel Hill	$777,976,677	6.00	138	72	25	56	14	$3,783,545
University of Wisconsin- Madison	$1,123,501,000	18.00	386	167	157	63	7	$94,170,000

*Data obtained from the fiscal year 2013 AUTM report.

**Table 2.** University of Kentucky research commercialization activity, 2010–2013 *.

Commercialization Activity	2010	2011	2012	2013
Disclosures	57	59	83	58
Patent Applications	28	22	12	17
Patents Issued	28	26	29	30
Licenses/Options Executed	9	8	9	9
Start-ups	6	7	6	3
License Income	$2,161,743	$1,544,664	$1,628,264	$4,800,000

*Data obtained from the fiscal year 2010–2013 AUTM reports.

The study herein was designed as a supplement to the previous study at the University of Kentucky ^11^ and focused on obtaining a more detailed understanding of the impediments to commercializing research at the university from the perspective of three faculty members that have been successful in the continuum of commercialization through successfully obtaining patents, licensing intellectual property and forming start-up companies. The rationale for conducting this supplemental study with only successful academic entrepreneurs was that we believed that more focused and individualized conversations with such entrepreneurs could provide more insight into the commercialization process versus conducting the study with individuals that have had more limited or no experience in commercializing research.

## Methods

The study herein is a supplement to and modeled closely after a similar, larger scale study conducted at the University of Kentucky specifically among cancer researchers ^11^. The methodology and design of the study was qualitative in nature and was based on two modules: an online survey (included as Supplementary materials S1) followed by a face-to-face interview. It is important to note that the prior study ^11^ did not include a face-to-face interview and was conducted with faculty that had both successfully commercialized their research and those that had not. It is also noteworthy that the respondents for this supplemental work span different research categories as defined in Table 4. The purpose of this supplemental research was to obtain more detailed information, primarily through the face-to-face interview, on the impediments to the commercialization of research at the University of Kentucky.

The selection criteria for inclusion in the study was that the selected participants must be faculty members, have active research programs and be successful academic entrepreneurs based on having obtained patents, licensed intellectual property and created start-up companies. The research subjects for this study were identified through searches of publically available databases containing information on the selection criteria. For module one, data were collected and managed using the Research Electronic Data Capture (REDCap) tool. REDCap is a secure, Internet-based study-support application ^12^. Module two data were recorded in written format during the face-to-face interview.

There are several limitations associated with this study. As a limited case study designed as a supplement to prior research ^11^, the results may not be translatable to other situations or research questions beyond that addressed in the original study ^11^, and the opinions of the three respondents may not be representative of all the stakeholders involved in the commercialization landscape at the University of Kentucky or elsewhere. Thus, the findings may not be generalizable to all faculty either at the University of Kentucky or at other universities, and the findings may or may not be capable of being generalized to other research areas. Further, as a cross-sectional study, the barriers experienced by the participants outside of the data collection window may not have been captured. Subject selection bias, which could lead to data and outcome bias, may also exist. Lastly, the study was designed to identify general challenges, thus more specific challenges were likely not captured by this analysis. Despite these limitations, the data obtained from this study, especially from the face-to-face interviews, provide additional supplemental information that enhances the findings of the previous study ^11^. Thus, this supplemental work provides more detailed information that can be presented to the administrative leadership of the commercialization process at the University of Kentucky.

This study was determined to not require review by the University of Kentucky Institutional Review Board. The research subjects consented to participate in the study electronically via engagement with the online survey and chose to participate in both modules of the study. The participants chose to remain anonymous beyond interaction with the investigators involved in the study.

## Results

### Professional productivity and commercialization perspective

The first series of survey questions, summarized in Table 3, aimed to assess the subjects’ category of research, professional productivity and their perspective on research commercialization. Respondent 1 classified his research as “translational;” he feels satisfied with his level of professional productivity in terms of publishing research manuscripts, obtaining grant funding and other means of academic productivity; and he indicated that he intends to commercialize additional research in the future. Despite believing that research commercialization is important in the academic setting and that his research field values such work, he feels that the University of Kentucky places little emphasis on and thus does not greatly value research commercialization. Respondent 2 classified his research as “basic;” he feels that his research is underutilized; and he intends to continue commercializing his work. Further, respondent 2 believes commercialization is important in an academic setting, yet the University of Kentucky does not emphasize such activity and he believes that his research field does not place an emphasis on commercialization. Respondent 3 classified her research as “basic/translational;” she is satisfied with her level of professional productivity; and, interestingly, despite having developed intellectual property and starting a company, she indicated that she may not pursue the commercialization of her research again in the future. Similar to respondent 1, respondent 3 also feels that research commercialization is important in the academic setting and that her research field values such activity, but that the University of Kentucky does not value research commercialization.

**Table 3.** Professional productivity and commercialization perspective.

Question	Respondent 1	Respondent 2	Respondent 3
Which category best describes your research?	Translational	Basic	Basic/Translational
Do you feel that your research results are sufficiently utilized through the generation of publications, grants, and other forms of professional productivity?	Yes	No	Yes
Do you intend to commercialize your research in the future?	Yes	Yes	No
Do you think research commercialization is important to promote within an academic setting?	Yes	Yes	Yes
Do you think the University of Kentucky places an emphasis on academic research commercialization to faculty?	No	No	No
Do you think your research field places an emphasis on academic research commercialization?	Yes	No	Yes

### Impediments to research commercialization

The second set of survey questions, summarized in Table 4, addressed the research subjects’ perceived impediments to commercializing research. Respondent 1 believes that risk, lack of investors, commercialization infrastructure, unsupportive university and federal policies, and “other barriers not listed” prohibit his efforts to effectively and efficiently commercialize research. Respondent 2 feels that commercialization infrastructure, lack of importance to academia (i.e., lack of emphasis placed on commercialization by academia), and “other barriers not listed” are the impediments that inhibit his commercialization efforts. The barriers identified by respondent 3 include the presence of risk, lack of time, expense, lack of investors, insufficient infrastructure, unsupportive university policies, and lack of industry partners.

**Table 4.** Impediments to research commercialization.

Potential Barrier	Respondent 1	Respondent 2	Respondent 3
There are no barriers to commercializing research at the University of Kentucky	No	No	No
There is unwanted risk associated with commercialization	Yes	No	Yes
I lack the expendable time	No	No	Yes
There is excessive expense	No	No	Yes
There is a lack of investors	Yes	No	Yes
There is a lack of infrastructure including facilities and staff to help in the commercialization process	Yes	Yes	Yes
Unsupportive University policies, procedures and/or regulations	Yes	No	Yes
Unsupportive federal policies, procedures and/or regulations	Yes	No	No
There is a lack of industry partners	No	No	Yes
Limited or no commercial application of my research exists	No	No	No
There is a lack of importance to academia	No	Yes	No
There is a lack of importance to my field	No	No	No
There is a lack of benefit to society	No	No	No
I have no interest in commercialization	No	No	No
Other barriers not listed	Yes	Yes	No

In the face-to-face interview, respondent 1 indicated that the “other barriers” included major prohibiting factors such as the lack of university support/infrastructure in areas of market analysis, grant development, and navigating legal matters including conflict of interest and intellectual property ownership issues. Of these “other” items, we had anticipated that such factors could be captured under the commercialization infrastructure and/or policy categories of answer choices in the survey. Ultimately, respondent 1 indicated that infrastructure issues are the most significant factors that impede research commercialization at the University of Kentucky. The subject also discussed how some of these barriers are more challenging and more difficult to overcome and that he felt that the barriers he has encountered are different at other universities. Respondent 2 indicated three major factors that negatively impact commercialization at the university and those are: 1) a lack of an entrepreneurial culture at the university level which has eroded the interest faculty have in pursuing the commercialization of their work; 2) inhibitory commercialization policies and an unwillingness for those policies to be malleable to individual commercialization situations/circumstances; and 3) insufficient and inhibitory commercialization infrastructure. Respondent 3 described the biggest barriers to academic research commercialization as faculty’s lack of business knowledge and commercialization background. Interestingly, she considers it more the responsibility of each faculty member to drive any potential commercial aspect of their work rather than rely on resources and support that may or may not exist at the university level. Since the majority of faculty do not receive any training in business or commercialization areas, she feels that this hampers the overall commercialization activity on university campuses.

Similar to the previous study among cancer researchers ^11^, these data suggest that faculty members experience multiple barriers in the commercialization process at the University of Kentucky. Additionally, in comparison with previous studies ^4– 11^, the data may suggest that not all barriers are consistent or common between individual faculty members (for example, expense, time constraints, insufficient infrastructure, and lack of industry partnerships were the most common barriers experienced among University of Kentucky cancer researchers ^11^). And, some barriers appear to be more prohibitive than others.

### Factors that could enhance research commercialization

The final set of questions, summarized in Table 5, were meant to determine which impediments would need to be overcome in order to increase faculty participation in research commercialization. Respondent 1 indicated that the barriers in the commercialization process do not deter him from attempting to commercialize his research, however, he believes that reducing/mitigating all the potential barriers surveyed, other than addressing royalty pay to inventors, would enhance research commercialization activity at the University of Kentucky. The subject also indicated that he would utilize outside (off campus) commercialization resources to lower the barriers he faces in order to improve his commercialization efforts. Respondent 2 would also use off campus resources to enhance his commercialization efforts and he believes that providing faculty protected time for commercialization efforts, providing additional and more helpful information to faculty about how to commercialize research, increasing the financial support available to entrepreneurial faculty, enhancing the commercialization infrastructure on campus, and increasing the emphasis placed on research commercialization would improve the commercialization activity at the university. Similar to respondents 1 and 2, respondent 3 would use off campus resources to commercialize her research, and she feels that providing information on how to commercialize, providing financial support, improving commercialization infrastructure, revising university policies, and increasing links to industry would improve commercialization activity at the University of Kentucky.

**Table 5.** Factors that could enhance research commercialization.

Factor	Respondent 1	Respondent 2	Respondent 3
Offering protected time specifically for commercialization activities	Yes	Yes	No
Increasing information on how to commercialize	Yes	Yes	Yes
Increasing financial support	Yes	Yes	Yes
Better and/or more infrastructure including facilities and staff to help in the commercialization process	Yes	Yes	Yes
Revising university policies, procedures and/or regulations	Yes	No	Yes
Revising federal policies, procedures and/or regulations	Yes	No	No
Increasing links to industry	Yes	No	Yes
Increasing emphasis placed by academia and/or my research field on the importance of research commercialization	Yes	Yes	No
Greater personal benefits including more royalty pay	No	No	No
Greater societal benefits	Yes	No	No
Nothing would help	No	No	No

These data are similar to the feelings reported by cancer researchers ^11^ in which respondents believe that mitigating many factors would presumably increase commercialization activity. Not all respondents, however, completely agree on all the factors that are important to address.

## Conclusion

This case study investigated the mindset of three successful academic entrepreneurs at the University of Kentucky in relation to the status of the research commercialization process and in context with the university’s general commercialization activity. The general state of the institution’s commercialization activity is modest relative to its benchmark institutions and stagnant in growth over time. The research subjects identified several factors that generally impede research commercialization and the subjects agreed that mitigating many factors may increase commercialization activity. Infrastructure insufficiencies, a lack of an emphasis by the university on the importance of research commercialization, a low to nonexistent entrepreneurial culture on campus, inhibitory policies, and a lack of business and commercialization knowledge among faculty were highlighted as the most significant barriers. While generally fitting with the impediments found at other universities and among cancer researchers at the University of Kentucky ^4– 11^, the results suggest that not all barriers are common or consistent between faculty and that some impediments may be more prohibitive than others. It is likely that the barriers vary between and among disciplines and the barriers may further vary based on an individual’s general experience with the commercialization process.

These data can be shared with the University of Kentucky’s Intellectual Property Development and Technology Transfer Office and the Office of the Vice President for Research and used as a guide to make changes that will improve the research commercialization process. The research subjects’ comments regarding commercialization infrastructure, a stagnant entrepreneurial culture, inhibitory commercialization policies, and faculty’s lack of business/commercialization knowledge may be particularly important to address in order to enhance commercialization activity at the university. Additionally, similar work could be conducted at and among other institutions. For example, a survey similar to the one herein and that used in the prior study ^11^ could be incorporated into the yearly AUTM licensing survey in order to gauge, on a much broader scale, the impediments to academic research commercialization as well as to understand how other institutions are mitigating such impediments. Understanding how institutions that are highly successful in commercializing research mitigate barriers in the process would be greatly beneficial to institutions that have low to modest commercialization activity.

## Supplementary materials

**Supplementary materials 1**

Research Commercialization Survey

Copyright: © 2015 Vanderford NL and Marcinkowski E2015Data associated with the article are available under the terms of the Creative Commons Zero "No rights reserved" data waiver (CC0 1.0 Public domain dedication).
