# Supplementary material for: A Case Study of the Impediments to the Commercialization of Research at the University of Kentucky
Source: F1000Res. 2015 Aug 4;4:133. Originally published 2015 May 28. [Version 2] doi: 10.12688/f1000research.6487.2 (PMC4566417; doi:10.12688/f1000research.6487.2)
Supplement: Supplementary file 1 [file f1000research-4-7398-s0000.tgz › 6bd36f72-3414-4473-8d98-ba6c0bb03471.pdf]

Research Commercialization Survey

1) Which category best describes your research? (Check all that apply)
   - ☐ Basic
   - ☐ Clinical
   - ☐ Translational (ie, bench to bedside)
   - ☐ Population and/or behavioral science
   - ☐ Applied

2) Do you feel that your research results are sufficiently utilized through the generation of publications, grants, and other forms of professional productivity?
   - ◯ Yes
   - ◯ No

3) Do you intend to commercialize your research in the future?
   - ◯ Yes
   - ◯ No

4) Do you think research commercialization is important to promote within an academic setting?
   - ◯ Yes
   - ◯ No

5) Do you think the University of Kentucky places an emphasis on academic research commercialization to faculty?
   - ◯ Yes
   - ◯ No

6) Do you think your research field places an emphasis on academic research commercialization?
   - ◯ Yes
   - ◯ No

Please indicate if you consider the following to be general challenges impeding the commercialization of YOUR research discoveries at the University of Kentucky:

		Yes	No
7)	There are no barriers to commercializing research at the University of Kentucky	◯	◯
8)	There is unwanted risk associated with commercialization	◯	◯
9)	I lack the expendable time	◯	◯
10)	There is excessive expense	◯	◯
11)	There is a lack of investors	◯	◯
12)	There is a lack of infrastructure including facilities and staff to help in the commercialization process	◯	◯
13)	Unsupportive University policies, procedures and/or regulations	◯	◯
14)	Unsupportive federal policies, procedures and/or regulations	◯	◯
15)	There is a lack of industry partners	◯	◯
16)	Limited or no commercial application of my research exists	◯	◯

17) There is a lack of importance to academia	○	○
18) There is a lack of importance to my field	○	○
19) There is a lack of benefit to society	○	○
20) I have no interest in commercialization	○	○
21) Other barriers not listed	○	○

---

22) Do you think that the barriers to commercializing research at the University of Kentucky are different than those at other academic institutions?
○ Yes
○ No

23) Would you use outside resources to commercialize your research rather than using University of Kentucky resources?
○ Yes
○ No

Please indicate if the following measures would help you be more active in research commercialization at the University of Kentucky:

	Yes	No
24) Offering protected time specifically for commercialization activities	○	○
25) Increasing information on how to commercialize	○	○
26) Increasing financial support	○	○
27) Better and/or more infrastructure including facilities and staff to help in the commercialization process	○	○
28) Revising university policies, procedures and/or regulations	○	○
29) Revising federal policies, procedures and/or regulations	○	○
30) Increasing links to industry	○	○
31) Increasing emphasis placed by academia and/or my research field on the importance of research commercialization	○	○
32) Greater personal benefits including more royalty pay	○	○
33) Greater societal benefits	○	○
34) Nothing would help	○	○

35) If there are any additional remarks you would like to have noted concerning the University of Kentucky and the commercialization of its research, please do so: __________________________________

36) Would you like to consent to a follow-up face-to-face interview regarding your experience with research commercialization at the University of Kentucky?

◯ Yes
◯ No
