# Supplementary material for: A Case Study of the Impediments to the Commercialization of Research at the University of Kentucky
Source: F1000Res. 2015 Aug 4;4:133. Originally published 2015 May 28. [Version 2] doi: 10.12688/f1000research.6487.2 (PMC4566417; doi:10.12688/f1000research.6487.2)
Supplement: Peer review history

# Peer review: Referee response for version 2

**DOI:** 10.5256/f1000research.7398.r9824

**Published:** 2015-08-17

**Authors:** Scott Crick

**Affiliations:** 1 Licensing Associate, Office of Technology Management, Washington University, St Louis, MO, USA

Overall, this version is definitely an improvement over V1. The major weakness I see remaining is the lack of specific suggestions for improving the technology transfer process. The most ubiquitous complaints seemed to be regarding the infrastructure, and the lack of appreciation of the importance and support for commercialization. First, it would be nice to define what is meant by the infrastructure. This term is fairly vague and could reasonably be viewed as fairly all-encompassing. A narrowing definition would be useful. For example, is the infrastructure referring specifically to the Tech Transfer Office, which would suggest the office is perhaps understaffed? Without more clearly defining the complaint, positing possible mitigating improvements becomes more difficult. In short, I am going to recommend approval of the article, but I think it would be improved by including specific suggestions for overcoming perceived barriers to tech transfer.

I have read this submission. I believe that I have an appropriate level of expertise to confirm that it is of an acceptable scientific standard.

# Peer review: Referee response for version 2

**DOI:** 10.5256/f1000research.7398.r9964

**Published:** 2015-08-13

**Authors:** Evan Facher

**Affiliations:** 1 Enterprise Development, University of Pittsburgh, Pittsburgh, PA, USA

I would like to thank the authors for their specific responses to my initial comments and for clarifying many of these concerns with substantial changes to the text of the submission that both clarify the purpose of the study and add to the data collected.

My initial review focused on three main areas for consideration by the authors:

1. Conclusions based on the survey/interview of a single academic entrepreneur;
2. Expansion of perspectives on reasons for innovations not being commercialized and;
3. Increase engagement with academic entrepreneurs on campus that have been both successful and unsuccessful with bringing their ideas to market. 

With the new submission, I believe the authors have addressed my three suggested areas of focus. It is now much clearer to me that this work was a supplement to an initial study performed to understand commercialization on campus. Through edits to the manuscript this rationale/purpose is now understood as a follow-up rather than a total brand new analysis. As such, there was a certain methodology that was being continued from the initial work that clarified the “customers” they were focused on as being faculty members that have successfully commercialized research. The study also increased the number of individuals that were interviewed from a single individual to multiple on campus allowing for a broader set of perspectives than initially obtained. Further, the authors did add focus on the limitations with the study based on design and purpose allowing the reader to more clearly understand the approach.

In conclusion, I would approve publication of this new version of the manuscript. The only additional recommendation I would provide from a practical perspective is that when presenting these findings to the university administrators, the authors should also offer some solutions to the issues they have uncovered.  Problem solving will be viewed more favorably than purely problem identifying.

I have read this submission. I believe that I have an appropriate level of expertise to confirm that it is of an acceptable scientific standard.

# Peer review: Referee response for version 2

**DOI:** 10.5256/f1000research.7398.r9823

**Published:** 2015-08-13

**Authors:** Jessica Silvaggi

**Affiliations:** 1 UWM Research Foundation, Milwaukee, WI, USA

The goal of the authors was to document any impediments to the commercialization process at the University of Kentucky. The study was spurred by the findings that UK significantly underperforms when compared to peer institutions of similar size. A previous study was published in 2013 regarding this same topic at UK for the commercialization of cancer research. In this study, similar surveys were conducted with 3 entrepreneurial respondents as opposed to a larger cohort of cancer researchers in the previous UK study.

The updated information from 2 more respondents is appreciated, but ideally it would be nice to have a larger group than 3. Getting information from those that had a positive and a negative experience is helpful to make changes to the system. If the ultimate goal is to provide this feedback to the UK TTO in order to institute changes, more details would be helpful. Some aspects that I think would benefit from further detail are the following:

1. Interviews of the actual TTO employees? What is keeping the University from making any changes?
2. Some background information on what other universities have done to rebrand their TTO. There have been many stories regarding offices doing an overhaul and rebranding and trying new things. Table 5 is a good start in figuring out some areas that need more improvement. A section that details what other offices have tried would be useful to people in technology transfer. The suggestions listed in table 5 are fairly standard practices that TTOs must consider. Can you include suggestions of how other universities have for instance increased links to industry or how they have offered more information to researchers teaching them how to commercialize? What are the policies or regulations that are restrictive at UK? Would utilizing more interns/students/post docs as staff help in bringing in more disclosures or provide more hands to evaluate inventions?

Overall the authors claim that the goal is to present this data as a guide to the UK TTO to make changes. It would be helpful to provide suggestions rather than telling them these results which they likely already know and realize are a problem.  It will be more useful to also provide some options that have been tried by other universities rather than just pointing out the failures. With these additions I think the article would be much stronger and more useful to other TTO offices.

I have read this submission. I believe that I have an appropriate level of expertise to confirm that it is of an acceptable scientific standard, however I have significant reservations, as outlined above.

# Peer review: Referee response for version 1

**DOI:** 10.5256/f1000research.6961.r9270

**Published:** 2015-07-01

**Authors:** Evan Facher

**Affiliations:** 1 Enterprise Development, University of Pittsburgh, Pittsburgh, PA, USA

The study by Vanderford and Marcinkowski attempts to identify challenges to the commercialization of innovations discovered at their institute of higher learning, the University of Kentucky. The goal of this work is to improve the sluggish local climate for translation of discoveries by communicating the findings resulting from this effort back to administrative leadership with the hope that the identification of these impediments leads to real change. By deploying a survey and subsequent interviews the authors plan to generate learnings sufficient to form the basis of their recommendations.

Movement of research discoveries from academic institutes to the marketplace is important not only for these discoveries to have their societal impact but also to drive economic growth of a region. The juxtaposition of most new jobs created over the last two decades coming from startup companies and much innovation from academia serving as the impetus for these new entities hints at the regional criticality of a university being able to successfully translate its discoveries into products reaching the market.

While the Vanderford and Marcinkowski article has a solid thesis and very good intent, it is however limited (in this reviewers mind) by a handful of items. First, the conclusions the authors generate are based on the survey and interview of a single academic entrepreneur. The data achieved from this individual is certainly very valuable but it might not be reflective of the other individuals on campus that have had experiences with commercialization of university-based research. The challenges described by this individual on campus ring true of the commercialization issues found throughout other academic organizations; however due to the small sample size it is hard to draw solid conclusions for the University of Kentucky as a whole. As one of the aims of the study is to report findings back to the administration with the goal of impacting change, I would suggest that the senior leadership, while sympathetic to the challenges expressed by the authors, will not institute changes based on such a small set of data that may not accurately reflect the general experiences of its academic entrepreneurs in totality.

I would recommend that the authors attempt to increase the number of study subjects to enhance the power of their research. In addition to surveys/interviews with academics that have successfully commercialized their innovations on campus, I would strongly urge the authors to include a set of individuals that have had unsuccessful experiences as well. Learnings from this cohort might provide an additional set of data to further drive the goals of enhancing translation at their institute.

The second item I would suggest the authors to contemplate in their assessments is expanding their perspectives on reasons for an innovation not being commercialized. It seems as if the main focus of the surveys/interviews is on structural elements involved in the workflow for moving a university idea to commercialization. It is often that the main reason for the lack of translation has nothing to do with the internal processes for moving the opportunity forward but rather that the innovation never really addressed a true market need despite the solid academic research. As such, regardless of the view of commercialization risk, the number of investors, the supportiveness of university policies or facilities/staff to advance the process, the idea itself is not commercialized because it does not contain a value proposition for any outside entity to take hold of. It is imperative to identify a product-market fit for an innovation as not all concepts should become companies and not all ideas impact the market. A strong technology translation capability cannot make up for an opportunity without a direct connection to an existing customer pain-point, which is only identified through a process of customer discovery outside of the university’s walls.

Lastly, from a practical perspective, I would recommend that as part of the engagement with a larger set of academic entrepreneurs on campus (both successful and unsuccessful), the survey/interview deployed by the authors engage these individuals in soliciting programs, opportunities, efforts, and ideas to improve the existing stagnant innovation culture on campus. I believe by engaging these “customers” on campus in dialogue focused on solving the challenge, administrative support will be more easily achieved.

In conclusion, I believe the authors are on the right track and that their efforts have significant merit. I would urge them to continue their work, expand the sample size, examine a bit broader set of reasons why the problems exist and work with their respondents to improve the situation.

I have read this submission. I believe that I have an appropriate level of expertise to confirm that it is of an acceptable scientific standard, however I have significant reservations, as outlined above.

# Response

**Published:** 2015-08-04

**Authors:** Nathan Vanderford

**Affiliations:** University of Kentucky, USA

Dear Dr. Facher,

Thank you for your time and comments. Your critique has helped guide us through revising the article. We would like to directly respond to some of your comments. We would first like to clarify that this case study was designed and conducted as a supplement to the prior study looking at the barriers associated with cancer research commercialization at the University of Kentucky (we have made this clarification in the new version of the article). The prior study’s conclusions were based solely on the responses to the survey as individual interviews were not conducted. Thus, the intent of the current study was to collect more specific, supplemental information through interviews with “successful” entrepreneurs. Given this intent and the study’s supplemental nature, we have purposefully not incorporated new research questions into the current case study. We have, however, at your suggestion, expanded the sample size by two respondents and this generated a number of additional significant comments that are important to understand regarding the university’s low commercialization rate. We agree with your assertions that additional reasons for low commercialization activity likely exist and that expanding our research questions and sample size to include a mix of stakeholders involved in the research commercialization process (including faculty that have been unsuccessful at commercializing their research and staff/administrators of the commercialization process, etc.) would aid in uncovering other issues. We would like to note, however, that expansion of this current work would change the intent (described above) and design of our study. Therefore, we have maintained the overall intent and design of the study other than adding the additional respondents. We believe that this design will allow us to bring some additional closure to the prior study. Lastly, to offset additional concerns, we have included a description of the limitations of this case study in the methods sections.

In closing, we hope that you will review this revised version of the article in light of our changes based on your comments as well as those of the other two reviewers (we hope that you will read the other reviewers’ comments as well as our response to those reviews) and in relation to its intended purpose of being a supplemental component to the prior study at the University of Kentucky.

Sincerely,

Nathan L. Vanderford and Elizabeth Marcinkowski

# Peer review: Referee response for version 1

**DOI:** 10.5256/f1000research.6961.r9005

**Published:** 2015-06-17

**Authors:** Scott Crick

**Affiliations:** 1 Licensing Associate, Office of Technology Management, Washington University, St Louis, MO, USA

This study attempted to identify potential impediments to commercialization of research at the University of Kentucky. The authors point out that, according to data from AUTM, the University of Kentucky ranks near the bottom in a number of key metrics of commercialization when compared to its benchmark institutions.  The goal of the study was to identify barriers to commercialization. These findings will then be brought to administration in hopes of rectifying the problem.

I think there are several issues with this work that, if addressed properly, will greatly strengthen its impact and utility not only at UK but also at other university technology transfer organizations. 

The first issue I have with the work is the inclusion of only a single faculty member with entrepreneurial experience. The rationale given was that that person could provide more insight into the process as a whole.  Looking at the data referenced in Table 1, the most striking discrepancy between UK and benchmark institutions is the number of invention disclosures.  When the data are normalized to the number of invention disclosures per institution, UK appears to be making good use of the inventions that are disclosed to them. It seems to me that one of the major issues at UK is simply getting inventors. While I agree that the entrepreneurial faculty member could have a lot of insight into issues after the first step, I wonder how much insight this person provided with regards to why people are not disclosing inventions. I am certain it would be possible to identify faculty that publicly disclosed potentially valuable assets without ever filing an invention disclosure, and I would suggest understanding those issues are 1) extremely important for improving commercialization at UK, and 2) more easily addressable at an institutional level than some other potential barriers.

The second issue with relying on input from a single faculty member is that barriers are variable (as even mentioned in the article) not only between individuals, but between disciplines and sub-disciplines as well.  Although the individual faculty member can identify barriers he/she has experienced, it would be disingenuous to suggest these barriers and the relative weight given to each of them is an accurate reflection of the system as a whole. A minor point....It would also be very helpful to know general field of research of the faculty member interviewed. "Translational" is very broad. Is he/she in pharma, biotech, medical devices, engineering...?

I have two recommendations to strengthen this article. 

My first recommendation is that the authors include in the case study at least two other faculty members with technology commercialization experience (not necessarily on par with the initial interviewee). I would also suggest that these faculty be from distinct research areas and departments. Although still qualitative, it would be insightful to see if these people with different types of technology, different department makeup, and very likely different experiences and backgrounds still identify the same barriers.

My second recommendation is to limit the scope to barriers that are perceived after invention disclosure. It appears as though there is an issue at UK (which I should say is certainly not unique) that I suspect has to do with education of potential inventors such that a number of these people are not even considering that their technology might have commercialization potential. A follow-up study trying to tease apart this issue would be interesting and may have broader appeal.

I have read this submission. I believe that I have an appropriate level of expertise to confirm that it is of an acceptable scientific standard, however I have significant reservations, as outlined above.

# Response

**Published:** 2015-08-04

**Authors:** Nathan Vanderford

**Affiliations:** University of Kentucky, USA

Dear Dr. Crick,

We thank you for taking the time to review our case study. Your critique has helped shape our current version of the article. We would like to respond to some of your comments. First, per your suggestion, we have expanded our sample size by two respondents. This expansion lead to the collection of a significant amount of additional crucial comments that are important to understand about the issues related to the low commercialization activity at the University of Kentucky. Second, in regard to your question about the respondents’ field of research, we understand your desire to have more information regarding the respondents’ research areas, but we feel that for confidentiality purposes, we cannot be any more specific; given the small sample size of “successful” academic entrepreneurs at the University of Kentucky, reporting a respondent’s specific research field could potentially allow for the identification of the subject. Next, you made very insightful comments regarding the desire to better understand the issues involved in the university’s low invention disclosure rate. We agree with your points and we would also like to address this issue. With that said, however, we would like to clarify that this case study was designed and conducted as a supplement to the prior study looking at the barriers associated with cancer research commercialization at the University of Kentucky; the sole intent of the current study was to collect more specific, supplemental information through interviews with the respondents (we have now explicitly stated this intent in the new version of the article). Given this intent and the study’s supplemental nature, we have purposefully not incorporated new research questions into the current case study. We feel that expanding the current study would likely change its primary intent. We do hope to address additional research questions, including understanding issues related to the low disclosure rate, in future work. Lastly, to offset additional concerns, we have included a description of the limitations of this case study in the methods sections.

In closing, we hope that you will review this revised version of the article in light of our changes based on your comments as well as those of the other two reviewers (we hope that you will read the other reviewers’ comments as well as our response to those reviews) and in relation to its intended purpose of being a supplemental component to the prior study at the University of Kentucky.

Sincerely,

Nathan L. Vanderford and Elizabeth Marcinkowski

# Peer review: Referee response for version 1

**DOI:** 10.5256/f1000research.6961.r8827

**Published:** 2015-06-05

**Authors:** Jessica Silvaggi

**Affiliations:** 1 UWM Research Foundation, Milwaukee, WI, USA

The goal of the authors was to document any impediments to the commercialization process at the University of Kentucky. The study was spurred by the findings that UK significantly underperforms when compared to peer institutions of similar size. A previous study was published in 2013 regarding this same topic at UK for the commercialization of cancer research. In this study, similar surveys were conducted with one single entrepreneurial respondent as opposed to a larger cohort of cancer researchers in the previous UK study.

They report that the major issue impeding commercialization, according to the respondent, is the lack of appropriate infrastructure at the university. The results were apparently similar to those found in this 2013 paper. I found this topic to be of great interest in comparing the performance to schools of similar benchmarks. The results could be helpful to many institutions that are underperforming and looking to boost commercialization. The survey questions used could be useful for any institution to keep track of the satisfaction of the researchers in regard to commercialization of their technologies, and to assess the adequacy of the performance of the technology transfer office.

Major concerns/Revisions

1. While I think the survey questions used were reasonable, I have doubts about the validity of asking a single entrepreneur the survey questions. It comes across as if the same 2013 study was repeated, but with only one person. I don’t feel that one person is a significant number for this type of paper to make conclusions with. N=1 doesn’t seem to be a true scientific sampling. Unfortunately, if more respondents were utilized, then this paper would appear to be a repeat of the earlier study. Perhaps another angle would be to survey the staff at UK as to why they are underperforming. Asking the tech transfer professionals and other research administrators would also be informative and provide a different angle to the original survey of cancer researchers. Or perhaps a broader survey of other departments would be helpful, rather than only focusing on the cancer department.
2. I feel that several other angles could have also been explored in this paper to contrast or follow up on the work in the 2013 paper about UK. After the first study were any changes made based on the conclusions? Did the number of staff change? Did any of the infrastructure change at UK? Did UK do anything to increase the level of commercialization? There are many other interesting questions that could be explored rather than repeating the study over again with one participant.
3. If the university has not responded to the previous study in any fashion, I find that of great interest.  Why hasn’t the technology transfer office been expanded and further supported? Why is there no incentive by those in charge of this area to revamp the office? There has been a large push at many universities to further promote entrepreneurship in faculty and students and support commercialization. In some online information it states that in the past UK was pushing to be a top 20 university by 2020. I am curious to know why there has been no change in the recent years.

Minor Revisions

1. When comparing benchmarks, another important factor missing in the table is the amount of research dollars. There is a ratio of expected disclosures per millions of dollars of research which varies, but is quoted in several locations at 1 disclosure per $1.5M-$3M and 1 start-up company per ~$100M. Knowing the amount of research dollars going into UK would help to show a lack of productivity with inventions at the university.  This information should be available through AUTM if not through the technology transfer office directly. Based on some online information the research expenditures at UK appear to be in the hundreds of millions of dollars. Assuming ~$400M in expenditures, one would expect about 130 disclosures. The UK website cites 84 disclosures in 2014.

Summary:

I feel that major revisions are necessary for this paper to add some new information and expand the scope of the study. The results here do not seem to add onto those previously found but simply confirm the previous findings with the survey of one entrepreneurial researcher. A different sample of respondents would greatly enhance this paper such as asking researchers from all departments, or focusing on the staff involved in technology transfer, commercialization and entrepreneurship. Or perhaps if changes were made by the tech transfer office and these did not have an effect, this would be good to know for other offices. There appears to be something missing from this story. This paper would be of interest if revamped to add a new twist distinct from the 2013 paper.

I have read this submission. I believe that I have an appropriate level of expertise to confirm that it is of an acceptable scientific standard.

I have read this submission. I believe that I have an appropriate level of expertise to confirm that it is of an acceptable scientific standard, however I have significant reservations, as outlined above.

# Response

**Published:** 2015-08-04

**Authors:** Nathan Vanderford

**Affiliations:** University of Kentucky, USA

Dear Dr. Silvaggi,

We greatly appreciate the time you dedicated to review our article. We found your comments very helpful as we revised the article. We are happy that you found this topic of interest and applicable to other institutions. Before reading the new version, we would like to address some of your comments and concerns. First, we appreciate your concerns regarding the “n-of-1” design of the study. As suggested by each reviewer, we have added two additional respondents to the study and this did indeed generate several additional important points regarding the issues related to the low commercialization activity at the University of Kentucky. Second, we appreciate your comments regarding how this work could be construed as a repeat of the prior study. We agree with your assertions that other issues are likely involved in the low commercialization activity at the university and we agree that surveying a mix of stakeholders involved in the research commercialization process could aid in uncovering other issues. However, we would like to clarify that this case study was, in fact, specifically designed and conducted as a supplement to the prior work looking at the barriers associated with cancer research commercialization at the University of Kentucky (we have made this clarification in the new version of the article). The prior study’s conclusions were based solely on the responses to the survey and individual interviews were not conducted. The intent of the current study was to collect more specific, supplemental information through interviews. Given this intent and the study’s supplemental nature, we have purposefully not incorporated new research questions into the current case study. We believe that future work would best address many of your comments which add additional research questions that would help understand other issues that may be connected to the low research commercialization activity. You have also made several important comments regarding whether the university has made any changes in the research commercialization process since the 2013 study. In fact, few changes have been made and that is one reason why it was important to conduct this case study; it was important to obtain specific comments from “successful” entrepreneurs so that these individuals could identify specific issues in the system. Finally, to offset additional concerns, we have included a description of the limitations of this case study in the methods sections. Per your minor point, we have now also added research expenditures to Table 1.

In closing, we hope that you will review this revised version of the article in light of our changes based on your comments as well as those of the other two reviewers (we hope that you will read the other reviewers’ comments as well as our response to those reviews) and in relation to its intended purpose of being a supplemental component to the prior study at the University of Kentucky.

Sincerely,

Nathan L. Vanderford and Elizabeth Marcinkowski
